# Variation in percentage weight bearing with changes in standing posture during water immersion: implication for clinical practice

**DOI:** 10.1186/1471-2474-15-261

**Published:** 2014-08-04

**Authors:** Babatunde OA Adegoke, Ajediran I Bello, Ademola O Abass

**Affiliations:** 1 Department of Physiotherapy, College of Medicine, University of Ibadan, Ibadan, Nigeria; 2 Department of Physiotherapy, College of Health Sciences, University of Ghana, Accra, Ghana; 3 Department of Human Kinetics, Faculty of Education, University of Ibadan, Ibadan, Nigeria

**Keywords:** Weight bearing, Hydro pool, Body weight, Standing posture

## Abstract

**Background:**

The degree of weightlessness during water immersion is usually estimated through percentage weight bearing (PWB). However, variations in PWB in different standing postures have not been documented. The study was designed to investigate the PWB of apparently healthy individuals in four standing postures at the anterior superior iliac spine level of immersion.

**Methods:**

One hundred and ninety-three consenting undergraduates were purposively enlisted in this study. Participants’ body weight (BW) was measured on land as well as in Erect Standing (ES), Grasp-Inclined-Prone-Standing (GIPS), Half-Grasp-Inclined-Towards-Side Standing (HGITSS) and Inclined-Standing with Head Support (ISHS) postures in hydro pool, using a specially designed water-proof weighing scale. PWB was calculated by dividing BW in water by BW on land and multiplying by 100. Data were analyzed using mean, standard deviation and ANOVA at α = 0.05.

**Results:**

The mean age and BW (on land) of the participants were 22.4 years and 60.7 kg respectively. Participants’ PWB were significantly different (p < 0.05) across the four standing postures. PWB was highest in ES and lowest in ISHS; PWB in ES (52.3 ± 5.8) being significantly higher (p < 0.001) than that observed in the derived standing postures. Further, PWB in GIPS (43.3 ± 5.6) and ISHS (43.2 ± 7.3) were significantly lower than in HGITSS (47.4 ± 5.2) posture while PWB in GIPS and ISHS postures were not significantly different (p > 0.05).

**Conclusion:**

Changes in standing posture have significant effect on PWB in hydro pool. The finding has implication for partial weight bearing exercises in hydro pool.

## Background

The use of water as a medium for therapy and recreation has many advantages due to its wide range of applications. Erect standing in water at rest is hence commonly chosen for weight bearing exercises and relaxation during recovery from bouts of exercise performance. However, most exercises in water have been found to be influenced by depth of water and body postures of the immersed individuals with profound impacts on axial loading of the musculoskeletal system [1]. These factors are reported to be crucial for effective prescriptions and applications of hydrotherapy. Although erect standing posture in water has been reported to be therapeutically useful in sports and clinical practice [2-7] information about the impact of changes in standing posture during water immersion on the effectiveness of the water medium is not yet documented.

Percentage Weight Bearing has been defined as the body weight in water divided by body weight on land and expressed in percentage [8-10]. The derived figures are often employed to estimate the amount of body weight borne by individuals during immersion in hydro pool. Previous researchers have been able to report variation in percentage weight bearing (PWB) of apparently healthy individuals in erect standing posture during graded immersion. Harrison and Bustrode reported PWB of a group of adults submerged to the seventh cervical vertebra (C-7), xisphisternum and anterior superior iliac spine (ASIS) to be 5.9%-10%, 25%-37% and 40%-56% respectively [8]. Similarly, Harrison *et al.* reported that participants in their study bored 25%, 25-50% and 50-75% of their body weight during static standing immersion to the levels of the clavicle, mid-trunk and groin respectively [9]. An apparent limitation of previous studies is the dearth of information about the potential influence of adjustment/variation of the standing posture at each specific level of immersion.

Variation of the fundamental standing posture, which might have implication for joint loading in an individual, is sometimes necessary in the course of hydro pool therapy. Postures that are variants of human fundamental positions (lying, sitting, kneeling, standing and hanging) are referred to as derived postures. They are obtained by changing the position of the head, arms or legs from that in the fundamental postures. Four standing postures have been described for use in hydrotherapy procedure but have not been investigated for their possible influence on axial joint loading during water immersion [11]. These are grasp-inclined-prone standing, half-grasp-inclined-towards-side standing, half-grasp-inclined-away-side standing and inclined-standing with head support. In view of the usefulness of these postures, there is a need to ascertain their impact on PWB in apparently healthy adults at a specific level of immersion. We hypothesized that changes in standing posture would have no significant influence on PWB of immersed adults across the four standing postures at the anterior superior iliac spine level of immersion.

## Methods

### Participants

Participants were apparently healthy consenting university undergraduates who met the following criteria: no skin rashes or open infection, absence of structural deformities affecting lower and upper limbs, and no medication that can negatively affect wakefulness/alertness during water immersion. They were enrolled into the study through purposive sampling technique.

### Instruments for data collection

**Figure 1:**
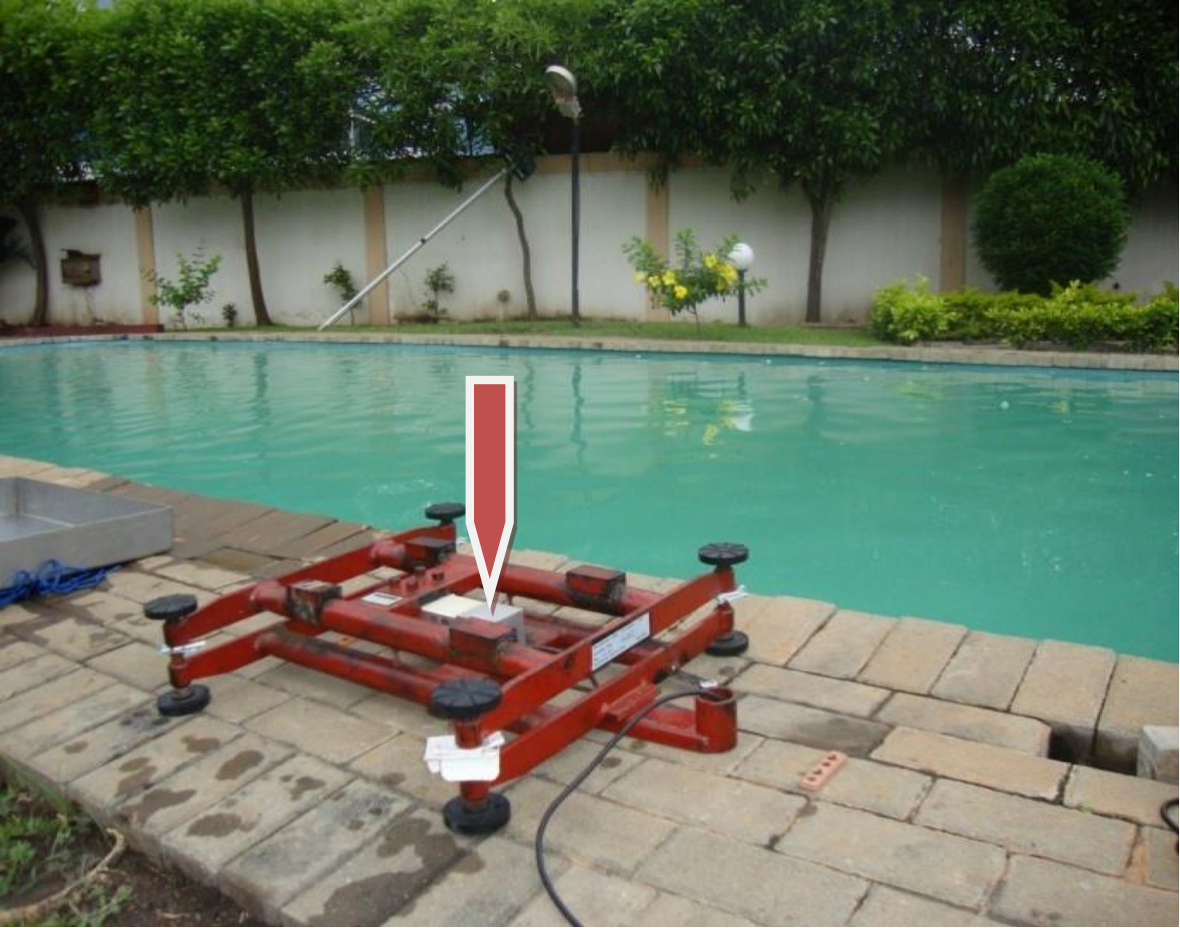
**Load cell within metal framework of the weighing scale (Red Arrow).**

**An industrial scale,** calibrated from 0-300 kg was used to measure the body weight of the participants both on land and in water. The scale operates on a water proof load cell IP67 which was converted to derive its power supply from an in-built rechargeable dry cell battery for the purpose of this study. It could last an average of eight hours when fully charged and measures body weight to the nearest 0.01 kg. The load cell is incorporated into a framework of rigid iron bars (Figure 1: Red arrow showing the load cell as the sensor of the body weight within the rigid iron bars from which load is transmitted electronically to the monitor outside the pool) that are caged in a removable large steel platform (60 cm × 60 cm) on which the participants stood. The measured weight is electronically transmitted into a digital indicator (5000 series Xtreme W) through a connecting cable of about 1.8 meters long so it can be positioned separately while the scale is immersed in the pool. The values of the measured weights were read and recorded by one of the Research Assistants.

**Wooden platforms** (Breath = 44 cm; Length = 36 cm; Height 15 cm) were specially designed and used to ensure accurate immersion to the level of anterior superior iliac spine. Each platform was wedged underneath with flat metal (10 kg) and perforated horizontally on its top to enable it to sink to the bottom of the pool.

### Protocols

Approval for this study was obtained from the Ethics Committee of the University of Ghana School of Allied Health Sciences. The informed consents of the participants were sought and obtained by requesting them to sign the consent forms after briefing them about the protocols and the potential risks/danger involved in the study. Participants were enrolled into the study through purposive sampling technique. The physical health status of the participants was ascertained through physical assessments to rule out limb length discrepancy which might affect balanced standing on the weighing scale as well as the presence of skin infection to avoid contamination of the pool. Female participants were specifically interviewed if they were undergoing their menstration so as to exclude them during the period. Participants who were under 18 years of age were excluded from the study.

The format for measeurement procedures were standardized on land and in water. All participants dressed in light swimming costumes which were dry-cleaned after each use to prevent cross infection. The body weights of participants were measured on land in erect standing while they stood barefooted on the weighing scale. The scores obtained on land in this posture served as a reference body weight with which the measured body weights obtained from other standing postures during immersion were compared.

Immersion into the pool was preceded by routine shower-bath by the reseachers and participants. The temperature range of the pool was between 26.5°C and 35°C (Critical temperature range) with an average of 29°C daily. Due to the variation in the heights of the participants, immersion to the anterior superior iliac spine was ensured by mounting wooden platforms on the weighing scale. Following accurate determination of the desired level, the body weight scores were read on the indicator at the pool edge whilst each participant assumed the following required standing postures: a. erect standing, during which the participants stood erect in fundamental position; b. grasp-inclined-prone-standing, in which participants stood and inclined anteriorly by grasping an object for support with both upper extremities fully extended; c. half-grasp-inclined-towards-side standing, in which participants stood and inclined sideway by grasping an object for support with a fully extended upper extremity; and d. inclined-standing with head support, in which participants stood and inclined posteriorly by leaning on an object for support using occiput of the head. After each measurement procedure for a participant, the weight of any mounted wooden platform was subtracted from the overall weight score to obtain the absolute body weight of the participant in all the four standing postures. Percentage weight bearing (PWB) was calculated by dividing the body weight of the participants in water by their body weight on land and multiplying by 100. The procedure adopted was based on the protocol described in the previous studies [8-10].

### Data analysis

Data were analyzed using the Statistical Package for Social Sciences (SPSS) version 19.0 Descriptive statistics of mean and standard deviation were used to summarize the participants’ age, height, body weight and percentage weight bearing. One-way analysis of variance (ANOVA) was used to compare participants’ percentage weight bearing across the four standing postures in water. Where ANOVA showed significant mean difference, Bonferroni post-hoc multiple comparisons analysis was used to test for specific differences between two mean variables. Alpha level was set at p < 0.05 for all the significant statistical tests with Bonferroni adjustment where it was found necessary.

## Results

One hundred and ninety-three apparently healthy adults (96 males, 97 females) participated in this study. The age of the participants ranged from 18 to 40 years (mean = 22.4 ± 2.7 years). The mean weight and height of the participants were 60.7 ± 10.4 kg and 1.6 ± 0.1 m respectively. The demographic characteristics are presented in Table 1. As expected, the participants’ body weight in all postures in the pool was lower than the body weight out of the pool. Percentage weight bearing of the participants were 52.3 ± 5.8 in erect standing, 43.3 ± 5.6 in grasp-inclined-prone standing (GIPS), 47.4 ± 5.2 in half-grasp-inclined-towards-side standing (HGITSS) and 43.2 ± 7.3 in inclined-standing with head support (ISHS) postures. There was a progressive decrease in PWB from ES to HGITSS, and further from GIPS to ISHS postures. ANOVA for PWB across the four postures showed significant F ratio at 0.05 level of significance (Table 2). Bonferroni post-hoc multiple comparisons indicated significantly higher PWB (p < 0.001) in ES (52.3 ± 5.8) posture than in all the derived standing postures. Further, participants had significantly higher PWB in HGITSS (47.4 ± 5.2) posture than in GIPS (43.3 ± 5.6) and ISHS (43.2 ± 7.3) postures (Table 3). However, there was no significant difference in the PWB of the participants in GIPS and ISHS postures.

**Table 1:** Demographic characteristics of participants

**Variable**	**Number**	**Minimum**	**Maximum**	**Mean (mean ± SD)**
Age (yrs)	193	18.0	40.0	22.4 ± 2.7
Height (m)	193	1.5	1.9	1.6 ± 0.1
Body weight (kg)	193	41.5	101.4	60.7 ± 10.4
BMI (kg/m^2^)	193	16.8	33.9	22.1 ± 3.4

**Table 2:** Summary of the analysis of variance (ANOVA) for percentage weight bearing of the participants in the four standing postures

**Mean PWB**	**Source**	**DF**	**SS**	**MS**	**F ratio**	**F prob**
52.3 ± 5.8 (ES) 43.3 ± 5.6 (GIPS)	Between group	3	1.061	0.354	96.10	0.001*
47.4 ± 5.2 (HGITSS)	Within group	768	2.826	0.004		
43.2 ± 7.3 (ISHS)	Total	771	3.887			

Legends: * = Difference is significant at p < 0.05.

Key: PWB = Percentage weight bearing; ES = Erect standing; GIPS = Grasp-inclined-prone standing; HGITSS = Half-grasp-inclined-towards-side standing; ISHS = Inclined-standing with head support.

**Table 3:** Summary of Bonferroni post hoc analysis for percentage weight bearing in four standing postures

**Posture**	**Mean Difference**	**p-value**
ES vs GIPS	9.0	<0.001*
GIPS vs HGITSS	4.1	<0.001*
GIPS vs ISHS	0.0	1.000
ISHS vs HGITSS	4.0	<0.001*
ES vs HGITSS	4.9	<0.001*
ES vs ISHS	8.9	<0.001*

Legends: * = Difference is significant at p = 0.012 i.e. (0.05/4).

The correlations between percentage weight bearing (PWB) and BMI, height and age of the participants were explored at anterior superior iliac spine using multiple linear regression. Results are presented in Table 4. PWB was significantly and inversely correlated with BMI in ES (β = -0.16; p =0.026), GIPS (β = -0.18; p = 0.014) and HGITSS (β = -0.14; p = 0. 048) postures. Participants’ PWB was also significantly and positively correlated with their height in ES (β = 0.21; p = 0.003) and HGITSS (β = 0.17; p = 0.021).

**Table 4:** Linear multiple regression showing relationship between socio-demographics and perecentage weight bearing of the participants

**Demographics**	**N**	**ES**		**GIPS**		**HGITSS**		**ISHS**	
		**β**	**p**	**β**	**p**	**β**	**p**	**β**	**p**
**BMI**	193	−0.16	0.026*	−0.18	0.014*	−0.14	0.048*	−0.11	0.140
**Height**	193	0.21	0.003*	0.12	0.094	0.17	0.021*	0.12	0.088
**Age**	193	0.02	0.067	−0.11	0.080	−0.14	0.096	−0.16	0.065

Legends: * = Difference is significant at p < 0.05.

However, PWB was not significantly correlated (p > 0.05) with BMI and height in ISHS posture. Similarly, PWB was not significant correlated (p > 0.05) with age in all the standing postures. The linear regression models for predicting PWB for male and female in all standing postures are as follows:

Percentage weight bearing in erect standing:

$$\mathrm{For}\;\mathrm{male},y=\;\left[0.722+(‒0.086\right)\mathrm{BMI}+0.0177\mathrm{Ht})$$

$$\mathrm{For}\;\mathrm{female},y=\left[0.781+(‒0.228\right)\mathrm{BMI}+\left(‒0.136\mathrm{Ht}\right)$$

Percentage weight bearing in Grasp-Inclined-Prone Standing:

$$\mathrm{For}\;\mathrm{male}\;y=[O.37+\left(‒0.11\mathrm{BMI}\right)+0.24\mathrm{Ht})$$

$$\mathrm{For}\;\mathrm{female}\;y=[1.12+\left(‒0.16\mathrm{BMI}\right)+\left(‒0.13\mathrm{Ht}\right)$$

Percentage weight bearing in Half-Grasp-Inclined-Towards Side Standing:

$$\mathrm{For}\;\mathrm{male}\;y=[O.52+\left(‒0.08\mathrm{BMI}\right)+0.23\mathrm{Ht}$$

$$\mathrm{For}\;\mathrm{female}:y=[1.06+\left(‒0.09\mathrm{BMI}\right)+\left(‒0.25\mathrm{Ht}\right)$$

Percentage weight bearing in Inclined-Standing-with Head Support:

$$\mathrm{For}\;\mathrm{male},\;y=[1.1+\left(‒0.09\mathrm{BMI}\right)+\left(‒0.01\mathrm{Ht}\right)$$

$$\mathrm{For}\;\mathrm{female}\;y=[O.65+\left(‒0.14\mathrm{BMI}\right)+\left(‒0.13\mathrm{Ht}\right)$$

Legends:

Ht=Height;BMI=BodyMassIndex

## Discussion

The degree of weightlessness of apparently healthy adults in four standing postures during immersion in water to anterior superior iliac spine level was investigated in this study. The mean percentage weight bearing (52.3 ± 5.8) obtained in the erect standing (ES) posture for this study falls within the range reported by the previous authors [8,9]. The similarity in the selection of the participants coupled with the conformity to the protocol in all the studies might have accounted for the consistent finding.

However, the results of this study did not agree with our hypothesis that variations in standing posture would not significantly influence the PWB. The assumption of four different standing postures indeed significantly affected the participants’ PWB while immersed to the anterior superior iliac spine level. Participants had significantly lower PWB in GIPS and ISHS postures than in ES and HGITSS standing postures, and significantly lower PWB in HGITSS than in ES postures. It thus implies that axial loading of weight bearing joints varies with different postures assumed in static standing with more weight being borne in erect standing than in the three derived standing postures. Conversely, PWB of the participants in GIPS and ISHS postures were not significantly different. The utilization of the outcomes of this result will be aptly informed by the goals of hydro pool exercises. Whilst GIPS and ISHS postures will be ideal for athletes who are recovering from bouts of sports participation or for relaxation, erect standing and half grasp inclined standing could be utilized during controlled weight bearing exercises in clinical practice.

Clinically, physiotherapists and sports medicine professionals find hydro pool useful as a package of therapeutic techniques either for rehabilitation or for general health promotion. Studies have established the potency of erect standing posture during immersion in enhancing recovery from bouts of strenuous exercises [2], enhancing fetal descent in pregnant women during labour process [3], allowing weight bearing moderation on the acute rheumatic or arthritic joints [4,6,7] and in patients recuperating from knee or hip arthroplasty [5]. The outcomes of this study could therefore provide wider options in the use of postures during hydro pool application.

Although, no literature is available with which to compare the results in the derived standing postures employed in the present study, the findings could be conceptually hinged on the effects of the alteration of body segments, causing non-alignment of the upward bouyant force with the downward gravitational force on the immersed individual [11-13]. The resultant turning effect (moment of buoyancy) from this disproportionate forces could cause displacement of line of forces beyond the center of bouyancy as well as the base of support of an individual thus influencing the amount of weight that would be transfered to the lower limbs [14]. This implies that, any movement or alteration of the limbs, trunk and head which alters the body’s shape whether above or below the surface of water will produce rotational effects, the amount of which depends on the degree of displacement, as will occur in any alteration in shape due to disability. Although, this submission is analogous to partial weight bearing during aided ambulation on land, the biophysical properties of water will expectedly enhance the biomechanics in the weight bearing joints.

Findings from this study indicate that the participants’ height and body mass index were significant predictors of percentage weight bearing (PWB). However, age was not a significant determinant of PWB in all standing postures when submerged to anterior superior iliac spine. It thus implies that age may not be important in determining PWB of the participants in all standing postures at this level of immersion. A similar study [9], however reported strong predictability of PWB in ES posture at ASIS by age, body weight on land and percent body fat. Although, the present study adopted different predicting variables from the previous study, age which is a common variable in both studies was not statitistcally significant in predicting PWB in the present study. Further discussion of findings on PWB in this study is hampered by the scarcity of literature on derived standing postures in research. From anectodal observation and search of literature, none of the inclined standing postures has been investigated for PWB apart from erect standing posture. Generally, there is an apparent dearth of research and policy on PWB for hydrotherapy uses in Sub-Saharan Africa. This situation thus calls for future efforts on this topic by sampling individuals with wide age gap.

### Limitation

The present study was limited by the high sensitivity of the adapted weight measuring scale used in this study. The water turbulence constantly caused transient fluctuation by a fraction of gram as read on the digital monitor placed by the pool side. As a result, the stable scores could only be read transiently within 2–5 seconds. The use of the specially devised force platform for measuring body weight under water could have been more appropriate.

## Conclusion

Within the limitation of this study, we concluded that the use of derived standing postures could appreciably influence axial loading of weight bearing joints of an immersed individual during water immersion to anterior superior iliac spine. The variation in the percentage weight bearing caused by different standing postures could potentially provide wider options for the use of hydro pool. Practitioners in Sports Medicine and Orthopaedics are therefore encouraged to embrace these findings whilst planning treatment program to enhance recovery from bouts of strenuous exercises and in rehabilitation of patients with arthritic condition to moderate weight bearing respectively. Future studies in this area are however required to substantiate these findings.

## Abbreviations

PWB: Percentage weight bearing; BW: Body weight; GIPS: Grasp-inclined-prone-standing; ES: Erect standing; HGITSS: Half-grasp-inclined-towards-side standing; ISHS: Inclined-standing with head support; ASIS: Anterior superior iliac spine; Ht: Height; BMI: Body mass index.

## Competing interests

The authors declare that they have no competing interests.

## Authors’ contributions

AIB: Prepared the first draft of the manuscript and contributed substantially to the execution of the research, development of research questions, the study design and the study protocol in general. BOAA: Contributed to the fine-tuning of the manuscripts, search for literature and overseeing the protocol of the research and transform the manuscript into an intellectual material. AOA: Also contributed in the management and analysis of data as well as the organization of the activities related to the study. All authors read and approved the final manuscript.

## Pre-publication history

The pre-publication history for this paper can be accessed here:

http://www.biomedcentral.com/1471-2474/15/261/prepub
